# LncRNA MEG3 inhibits the progression of prostate cancer by modulating miR‐9‐5p/*QKI‐5* axis

**DOI:** 10.1111/jcmm.13658

**Published:** 2018-11-22

**Authors:** Meng Wu, Yawei Huang, Tongchang Chen, Weichao Wang, Shiguang Yang, Zhenfeng Ye, Xiaoqing Xi

**Affiliations:** ^1^ Department of Urology The Second Affiliated Hospital of Nanchang University Nanchang Jiangxi China

**Keywords:** lncRNA MEG3, miR‐9‐5p, prostate cancer, *QKI‐5*

## Abstract

This study was designed to detecting the influences of lncRNA MEG3 in prostate cancer. Aberrant lncRNAs expression profiles of prostate cancer were screened by microarray analysis. The qRT‐PCR and Western blot were employed to investigating the expression levels of lncRNA MEG3, miR‐9‐5p and *QKI‐5*. The luciferase reporter assay was utilized to testifying the interactions relationship among these molecules. Applying CCK‐8 assay, wound healing assay, transwell assay and flow cytometry in turn, the cell proliferation, migration and invasion abilities as well as apoptosis were measured respectively. LncRNA MEG3 was a down‐regulated lncRNA in prostate cancer tissues and cells and could inhibit the expression of miR‐9‐5p, whereas miR‐9‐5p down‐regulated *QKI‐5* expression. Overexpressed MEG3 and *QKI‐5* could decrease the abilities of proliferation, migration and invasion in prostate cancer cells effectively and increased the apoptosis rate. On the contrary, miR‐9‐5p mimics presented an opposite tendency in prostate cancer cells. Furthermore, MEG3 inhibited tumour growth and up‐regulated expression of *QKI‐5* in vivo. LncRNA MEG3 was a down‐regulated lncRNA in prostate cancer and impacted the abilities of cell proliferation, migration and invasion, and cell apoptosis rate, this regulation relied on regulating miR‐9‐5p and its targeting gene *QKI‐5*.

## 1 INTRODUCTION

Prostate cancer (PCa) as one the most common malignancy in male around the world threatening men's health.^1^ Chen et al reported that PCa was the seventh in incidence and the tenth in mortality in malignant tumours in males in China.^2^ Approximately 10% of newly diagnosed patients showed evidence of locally advanced prostate cancer, and 5% showed distant metastasis.^3^ Therefore, achieving a comprehensive understanding of pathogenesis and identifying a novel bio‐labelling for early prediction and treatment in prostate cancer is urgently needed.^4^

More and more reports proved that long non‐coding RNAs (lncRNAs) impacted cell proliferation, apoptosis and tumour prognosis.^2^, ^4^ Resent reports proved that lots of lncRNA functioned as carcinogens or tumour suppressors in diverse tumours, including PCa.^5^ For example lncRNA SNHG1 promoted cell proliferation in prostate cancer.^6^ Besides, lncRNA MEG3 inhibited proliferation and metastasis of gastric cancer via p53 signalling pathway.^7^ However, the research around molecule mechanism of MEG3 in human prostate cancer still exist parts of unclear region, which excited us to exploring the influences of MEG3in the progression of prostate cancer.

MicroRNAs (miRNAs), belongs to a class of small non‐coding regulatory RNA molecules, also involved in many biological processes, such as cell development, differentiation, proliferation and apoptosis.^3^, ^8^ LncRNAs and miRNAs are thought to interact with one another to create an additional layer of regulational complexity.^9^ Ke et al revealed that silencing the lncRNA HOTAIR could inhibit malignant biological developments of human glioma cells through modulation of miR‐326.^10^ In addition, long non‐coding RNA TUG1 acted as a tumour promoting factor, could contribute to tumorigenesis of human osteosarcoma depending on regulating miR‐9‐5p/*POU2F1* expression.^8^ Furthermore, it has also been reported that MEG3 could bind to miR‐9 and regulated the expression of *E‐cadherin* and *FOXO1*.^11^ Therefore, we hypothesized that MEG3 might function as ceRNA for miR‐9‐5p in prostate cancer.

The Quaking (QKI) protein belongs to the signal transduction and activation of RNA (STAR) protein family and is a key post‐transcriptional regulator,^12^ which involved in several cancer regulatory mechanisms, including gastric cancer, myocardial ischaemia and oligodendrocyte.^13^ Recent findings indicated that genomic depletion of *QKI‐5* increases proliferation and dedifferentiation of cancer cells, which indicated that *QKI‐5* is a tumour suppressor for many cancer types.^14^ In our study, a series of experiments carried out that *QKI‐5* involved in regulating the development of prostate cancer, which was consistent with the previous reports.^15^

In this study, relied on detecting the down‐regulated lncRNA MEG3 in prostate cancer, the interactions among MEG3, miR‐9‐5p and *QKI‐5* were investigated and the influences on prostate cancer were explored subsequently. Hence, lncRNA MEG3 regulated miR‐9‐5p/*QKI‐5* axis in prostate cancer.

## 2 MATERIALS AND METHODS

### 2.1 Clinical specimens

In this study, 85 pairs of prostate cancer tissues and adjacent tissues were collected from patients who underwent surgical resection at the Second Affiliated Hospital of Nanchang University. All patients were confirmed by experienced pathologists and none was received preoperative radiotherapy or chemotherapy. This study was approved by the Ethics Committee of the Second Affiliated Hospital of Nanchang University. All the tissues were stored in liquid nitrogen at −80°C for further experiments.

### 2.2 Cell culture and treatment

Three prostate cancer cell lines PC‐3, DU145 and VCaP were cultured in the Dulbecco's Modified Eagle Medium (DMEM) (Invitrogen, Carlsbad, CA, USA) including 10% foetal bovine serum. The prostate cancer cell line 22RV1 was cultured in the Roswell Park Memorial Institute‐1640 medium (RPMI‐1640) (Gibco, Carlsbad, CA, USA). The prostate epithelial cell line WPMY‐1 was maintained in DMEM medium supplemented with 5% foetal bovine serum. Cells were purchased from the American Type Culture Collection (ATCC, Manassas, VA, USA). Lipofectamine™ 3000 (Invitrogen) was diluted with 250 μL DMEM medium. The cell transfection was followed by manufacturer's protocols. The carrier used in the experiment was pcDNA3.1. Cells were generally assigned to different groups as follows: (1) mock group and pcDNA3.1‐MEG3 group; (2) negative control (NC) group, miR‐9‐5p mimics group, miR‐9‐5p inhibitor group and miR‐9‐5p mimics+ pcDNA3.1‐MEG3 group; (3) NC group, miR‐9‐5p mimics group, pcDNA3.1‐MEG3 group and miR‐9‐5p mimics+ pcDNA3.1‐MEG3 group.

### 2.3 Microarray analysis

The microarray datasets have been deposited to Gene Expression Omnibus database (http://www.ncbi.nlm.nih.gov/geo, accession number GSE55909). The differential expression lncRNAs were screened using SurePrint G3 human lncRNA microarrays (Agilent). We identified the differentially expressed lncRNAs with a discriminating parameter of adjusted *P* value <.05 and fold change >2.

### 2.4 QRT‐PCR

Total RNA was isolated and reverse transcribed into cDNA according to M‐MLV Reverse Transcriptase (TaKaRa, Tokyo, Japan). And then real‐time PCR assay was proceed by SYBR Prime Script PLUS RT‐RNA PCR Kit (TaKaRa). The reaction condition of PCR is 95°C for 30 seconds, 62°C for 40 seconds and repeat the process for 40 times in sequence. The relative expression levels were counted by $2^{-\Delta \Delta C_{t}}$ method and StepOne software was employed to analysing the data. The internal reference we used was GAPDH, listed in Table 1 together with the primer sequence.

**Table 1:** Primer sequence

Primer	Sequence (5′‐3′)
MEG3 forward	5′‐CTGCCCATCTACACCTCACG‐3′
MEG3 reverse	5′‐CTCTCCGCCGTCTGCGCTAGGGGCT‐3′
miR‐9 forward	5′‐AGCTTGCTGCACCTTAGTCT‐3′
miR‐9 reverse	5′‐TGTGTGCGGCTAGAACATCC‐3′
QKI‐5 forward	5′‐TAGCAGAGTA CGGAAAGACAT‐3′
QKI‐5 reverse	5′‐GGGTATTCTT TTACAGGCACAT‐3′
GADPH forward	5′‐GACCTGACCTGCCGTCTA‐3′
GADPH reverse	5′‐AGGAGTGGGTGTCGCTGT‐3

### 2.5 Cell counting kit‐8 (CCK‐8) assay

The DU 145 cells were seeded in 96‐well plates for evaluating proliferation ability by CCK‐8 assay (Dojindo, Kumamoto, Japan). Cells were then cultured for 0, 1, 2, 3 or 4 days before addition 10 μL of CCK‐8 (5 mg/mL) to the culture medium in each well. The absorbance at 450 nm and measured it by Thermo‐max microplate reader (Thermo Fisher Scientific, Waltham, MA, USA).

### 2.6 Wound healing assay

First, DU 145 cells were seeded in 6‐well plates. When the cells reached confluence, the surface of the plates was lightly scratched using a sterile micropipette tip. The floating cells then was added with DMEM medium with 10% FBS. An inverted optical microscope (×200) (Nikon, Japan) was used to monitor the closure of the wound at 0, 24 and 48 hours. Each group repeated the process thrice.

### 2.7 Flow cytometry (FCM) assay

After transfection, trypsinization was used to harvesting cells, and then stained orderly with FITC‐Annexin V and PI according to the FITC‐Annexin V Apoptosis Detection Kit (BD Biosciences, San Jose, CA, USA). The cells were analysed with a flow cytometry (FACScan, BD Biosciences) equipped with a Cell Quest 3.0 software.

### 2.8 Transwell assay

The 24‐well transwell chambers (Costar, Corning, Switzerland) with Matrigel‐coated membranes were used for invasion assay. DU 145 cells were seeded on the RPMI 1640 medium and the invading cells in the lower chamber were stained by 0.1% crystal violet. The cells were observed under the phase contrast microscope (×200) (Nikon).

### 2.9 Luciferase reporter assay

The fragment of MEG3 containing the predicted miR‐9‐5p binding site was amplified by PCR. And then was cloned into a Dual‐luciferase miRNA Target Expression Vector (Promega, Madison, WI, USA) for forming MEG3‐wild‐type (MEG3‐Wt). The same approach was used to forming MEG3‐mutated‐type (MEG3‐Mut). Similarly, *QKI‐5*‐wild‐type (*QKI‐5*‐Wt) and *QKI‐5*‐mutated type (*QKI‐5*‐Mut) were set up. Then the luciferase activities were tested by Dual‐luciferase reporter assay system (Promega, Madison, WI, USA).

### 2.10 RNA immunoprecipitation (RIP) assay

RNA immunoprecipitation assay was performed by Imprint RNA immunoprecipitation kit (Sigma‐Aldrich, St. Louis, MO, USA) referring to the recommended protocols of manufacturer. Firstly, IgG‐induced chondrocytes were collected and resuspended in RIP lysis bufer (Solarbio), subsequently centrifuged at 12 000 *g* for 5 minutes. Ten, cell lysate was incubated with anti‐Argonaute2 (anti‐Ago2) or antiIgG (negative control) overnight at 4°C, followed by the addition of Protein A magnetic beads to get the immunoprecipitation complex. Total RNA was isolated using GenElute™ Total RNA Purifcation Kit (Sigma‐Aldrich). Lastly, the relative enrichment of MEG3 and miR‐9‐5p were determined by RT‐qPCR analysis.

### 2.11 Western blot

DU 145 cells were lysed in RIPA buffer. Proteins were separated on 10% SDS‐PAGE and electro‐transferred to nitrocellulose membranes. And then were incubated with anti‐*QKI‐5* (0.5 mg/mL, Boston Biochem, Cambridge, MA, USA) and anti‐GADPH (Boston Biochem) at 4°C overnight, and then incubated with a HRP‐labelled secondary antibody IgG (Boston Biochem) at room temperature for 3 hours. Immunolabelling was visualized using the ECL system (Amersham, Bucks, UK).

### 2.12 Xenograft mice model in vivo

The posterior flank of the 12 male BALB/c nude mice (5‐week‐old) were injected with 2 × 10^7^ DU 145 cells transfected with pcDNA3.1‐MEG3 or pcDNA3.1‐NC, subcutaneously. Tumour volumes were examined every 7 days and then calculated. The animal experiment was performed in compliance with the authenticated animal protocols of Ethical Committee of Animal Welfare of the Second Affiliated Hospital of Nanchang University.

### 2.13 Immunohistochemistry

The paraffin‐embedded xenograft tumour tissues were fixed and dewaxed, rinsed with PBS. Samples were incubated with primary antibodies anti‐*QKI‐5* (0.5 mg/mL, Boston Biochem) and GAPDH (Boston Biochem) at 4°C overnight, followed by a secondary antibody rabbit IgG (rabbit polyclonal; Cell Biotech, Tianjin, China) for 1 hour. The cells were incubated with 0.5 mg/mL of DAPI for 15 minutes at 37°C. All cover slips were examined under a spectral laser scanning confocal microscope (Nikon C1‐Si, Mississauga, Canada). Images were analysed using EZ‐C1 3.20 FreeViewer software.

### 2.14 Statistical analysis

Statistical analyses were performed with GraphPad Prism 6.0 software and data were expressed as mean ± SD. Statistical comparisons were made by one‐way analysis of variance (ANOVA) and *P* < .05 instructed a statistically significant difference.

## 3 RESULTS

### 3.1 LncRNA MEG3 was significantly down‐regulated in prostate cancer

**Figure 1:**
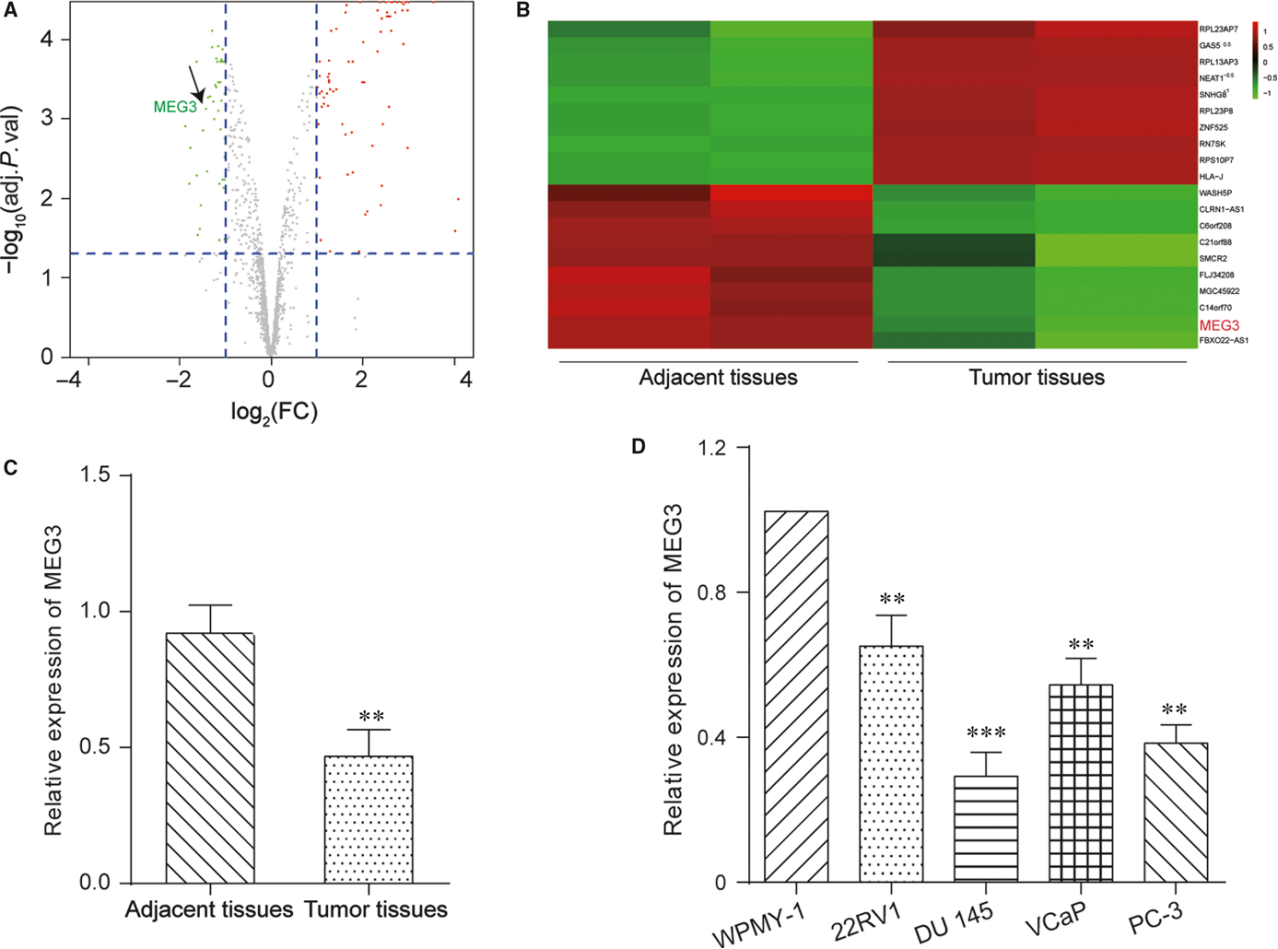
LncRNA MEG3 was down‐regulated in prostate cancer. A, The volcanic map showed some of the differentially expressed lncRNAs between prostate cancer tissues and adjacent tissues. B, The heat map showed lncRNA MEG3 was down‐regulated. C, The expression level of MEG3 was decreased in PCa tissues by qRT‐PCR. **P < .01, compared with adjacent tissues. D, The expression level of MEG3 was down‐regulated in prostate tumour cell lines (22RV1, DU 145, VCaP and PC‐3) by qRT‐PCR. **P < .01, ***P < .001, compared with WPMY‐1 cells

The differentially expressed lncRNAs were screened and showed in the volcano plot by microarray analysis (Figure 1A). The top 10 up‐ and down‐regulated lncRNAs were chosen to draw the heat map (Figure 1B) and MEG3 ranked second among these lncRNAs. The results of qRT‐PCR revealed that the level of lncRNA MEG3 was significantly decreased in prostate cancer tissues (Figure 1C). Meanwhile, a lower level MEG3 was obtained in the 4 prostate cancer cell lines (PC‐3, DU145, VCaP and 22RV1) compared with the human prostate epithelial cell line WPMY‐1 (Figure 1D). Besides that, the DU 145 cell line was used for the further experiments, the expression of MEG3 in DU 145 cell was the lowest.

### 3.2 LncRNA MEG3 inhibited prostate cancer

**Figure 2:**
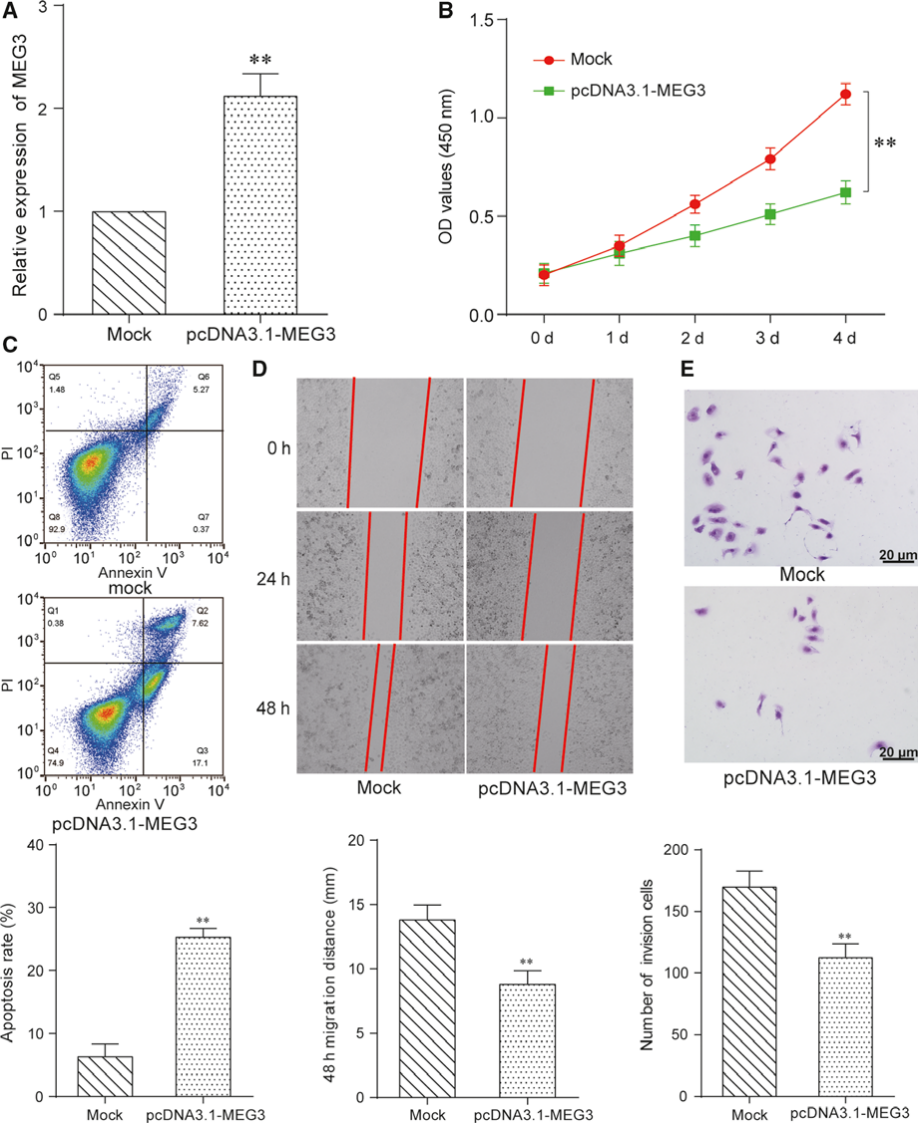
LncRNA MEG3 inhibited prostate cancer. A, QRT‐PCR results showed that the expression of MEG3 was up‐regulated in pcDNA3.1‐MEG3 group. **P < .01, compared with mock group. B, CCK‐8 assay showed pcDNA3.1‐MEG3 group inhibited DU 145 cells proliferation. **P < .01, compared with mock group. C, Cells apoptosis rate of pcDNA3.1‐MEG3 group increased by FCM assay. **P < .01, compared with mock group. D, Cells migration of pcDNA3.1‐MEG3 group was inhibited by wound healing assay. **P < .01, compared with mock group. (E) Transwell assay revealed that cells invasion of pcDNA3.1‐MEG3 group was depressed. **P < .01, scale bar = 20 μm, compared with mock group

MEG3 was up‐regulated in DU 145 cells when transfected with pcDNA3.1‐MEG3 (Figure 2A). CCK‐8 assay (Figure 2B) showed that pcDNA3.1‐MEG3 inhibited cell proliferation and FCM assay (Figure 2C) verified that pcDNA3.1‐MEG3 promoted cell apoptosis. Wound healing assay indicated that migration distance of DU 145 cells transfected with mock was farther, compared with that of DU 145 cells transfected with pcDNA3.1‐MEG3 (Figure 2D). PcDNA3.1‐MEG3 could also inhibit cell invasion according to transwell assay (Figure 2E). Therefore, pcDNA3.1‐MEG3 inhibited cell proliferation, migration and invasion while increased cell apoptosis rate. As a result, MEG3 inhibited prostate cancer.

### 3.3 MEG3 served as a sponge of miR‐9‐5p and inhibited prostate cancer

**Figure 3:**
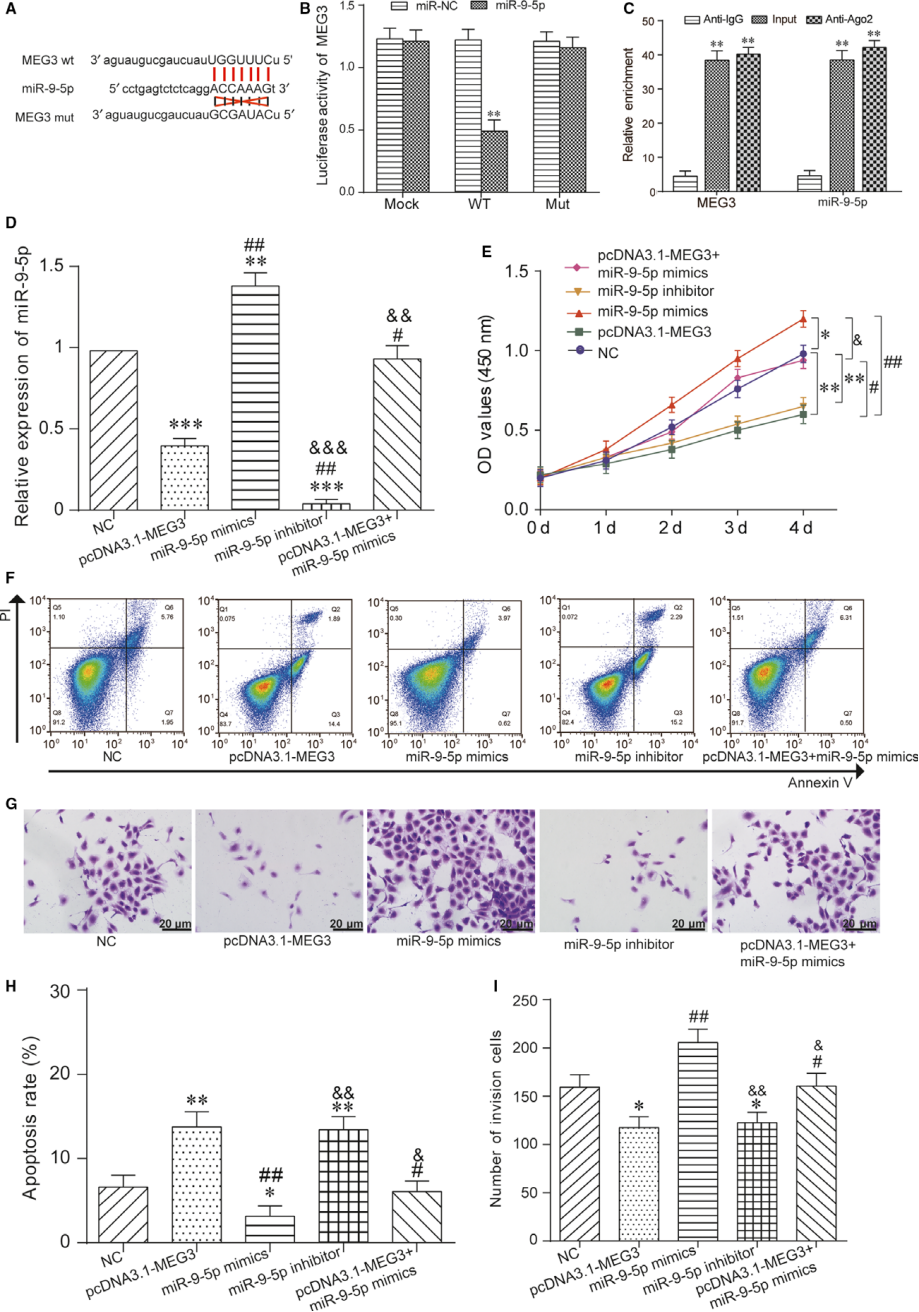
MEG3 served as a sponge of miR‐9‐5p and inhibited prostate cancer. A, The targeting relationship between MEG3 and miR‐9‐5p. B, The luciferase intensity of DU 145 cells transfected with MEG3 wild‐type and miR‐9‐5p was significantly reduced. **P < .01, compared with miR‐NC group. C, The interaction between MEG3 and miR‐9‐5p was detected by RNA immunoprecipitation (RIP) with Ago2 antibody. D, The expression of miR‐9‐5p in pcDNA3.1‐MEG3 group was reduced, whereas miR‐9‐5p mimics group was increased. The miR‐9‐5p expression level in the miR‐9‐5p inhibitor group was the lowest. **P < .01, ***P < .001, compared with NC group, ^#^ *P* < .05, ^##^ *P* < .01, compared with pcDNA3.1‐MEG3 group, ^&&^ *P* < .01, ^&&&^ *P* < .001, compared with miR‐9‐5p mimics group. E, CCK‐8 assay showed that the miR‐9‐5p mimics group exhibited higher cell proliferative capacity. Cell proliferative capacity of pcDNA3.1‐MEG3 group and miR‐9‐5p inhibitor group was decreased. *P < .05, **P < .01, compared with NC group, ^#^ *P* < .05, ^##^ *P* < .01, compared with pcDNA3.1‐MEG3 group, ^&^ *P* < .01, compared with miR‐9‐5p mimics group. F, H, FCM assay revealed that cell apoptosis rate of pcDNA3.1‐MEG3 group and miR‐9‐5p inhibitor group were increased, whereas miR‐9‐5p mimics group was decreased. *P < .05, **P < .001, compared with NC group, ^#^ *P* < .05, ^##^ *P* < .01, compared with pcDNA3.1‐MEG3 group, ^&^ *P* < .05, ^&&^ *P* < .01, compared with miR‐9‐5p mimics group. G, I, Cell invasion ability of pcDNA3.1‐MEG3 group and miR‐9‐5p inhibitor group were decreased, whereas miR‐9‐5p mimics group was promoted by transwell assay. *P < .05, compared with NC group, ^#^ *P* < .05, ^##^ *P* < .01, compared with pcDNA3.1‐MEG3 group, ^&^ *P* < .05, ^&&^ *P* < .01, compared with miR‐9‐5p mimics group. Scale bar = 20 μm

The targeting relationship between MEG3 and miR‐9‐5p was showed in Figure 3A. And then the luciferase reporter assay indicated that MEG3 served as a sponge of miR‐9‐5p. The results of luciferase reporter gene assay validated that cotransfected with MEG3‐wt and miR‐9‐5p obtained a weakened luciferase activity compared with the miR‐NC group and the MEG3‐mut group (Figure 3B). Furthermore, RIP assay was performed with Ago2 antibody to confirm the potentially endogenous interaction between MEG3 and miR‐9‐5p. The results revealed that MEG3 and miR‐9‐5p were largely captured by anti‐Ago2 compared with the negative control in IgG‐induced chondrocytes (Figure 3C). The results of qRT‐PCR verified that miR‐9‐5p expression level inpcDNA3.1‐MEG3 group was significantly reduced while was significantly increased in miR‐9‐5p mimics group. Besides, miR‐9‐5p was hardly expressed in the miR‐9‐5p inhibitor group. The miR‐9‐5p expression level of pcDNA3.1‐MEG3 & miR‐9‐5p mimics group was same as NC group (Figure 3D). CCK‐8 assay showed that the miR‐9‐5p mimics group exhibited higher cell proliferative capacity than other 4 groups. The cell proliferative capacity in pcDNA3.1‐MEG3 group was reduced, which was the same as miR‐9‐5p inhibitor group. Cell proliferative capacity of pcDNA3.1‐MEG3 & miR‐9‐5p mimics group was same as NC group (Figure 3E). The results of FCM assay revealed that cell apoptosis rate of pcDNA3.1‐MEG3 group and miR‐9‐5p inhibitor group were significantly higher than other 3 groups, whereas miR‐9‐5p mimics group was the lowest and pcDNA3.1‐MEG3 & miR‐9‐5p mimics group was same with NC group (Figure 3F,H). After transwell assay, cell invasion ability in pcDNA3.1‐MEG3 group and miR‐9‐5p inhibitor group was weakened, whereas the ability in the miR‐9‐5p mimics group was strengthened. The pcDNA3.1‐MEG3 & miR‐9‐5p mimics group was same with NC group (Figure 3G,I). Concluded from the above experiments, MEG3 served as a sponge of miR‐9‐5p to inhibit prostate cancer.

### 3.4 MEG3 served as a sponge of miR‐9‐5p and regulated *QKI‐5*

**Figure 4:**
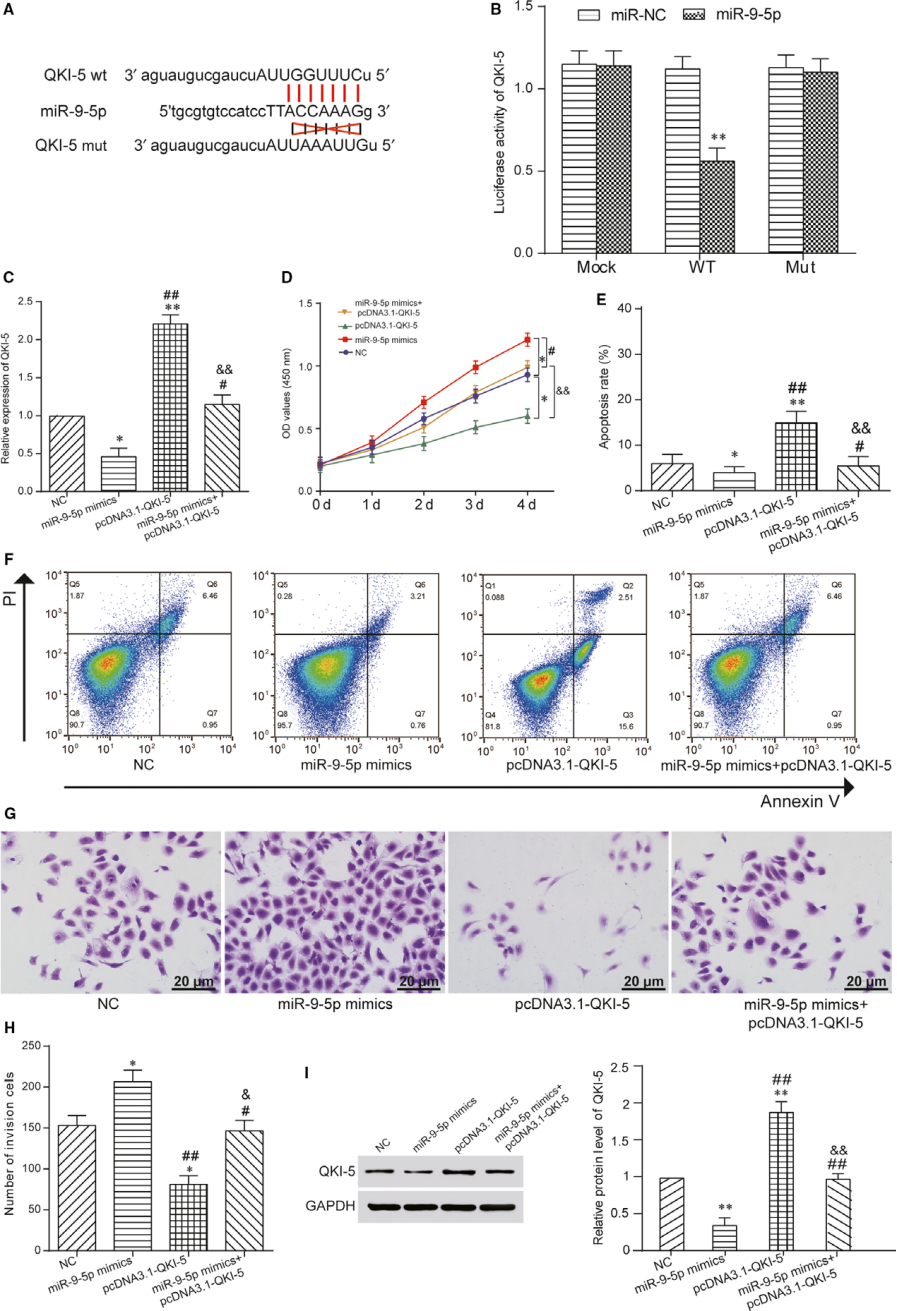
MiR‐9‐5p regulated expression of *QKI‐5*. A, The targeting relationship between miR‐9‐5p and *QKI‐5*. B, The luciferase intensity of DU 145 cells cotransfected with *QKI‐5* wild‐type and miR‐9‐5p was reduced. **P < .01, compared with miR‐NC group. C, The *QKI‐5* expression level of pcDNA3.1‐QKI‐5 group was higher, whereas miR‐9‐5p mimics group was reduced by qRT‐PCR. *P < .05, **P < .01, compared with NC group, ^#^ *P* < .05, ^##^ *P* < .01, compared with miR‐9‐5p mimics group, ^&&^ *P* < .01, compared with pcDNA3.1‐ QKI‐5 group. D, The cell activity of miR‐9‐5p mimics group increased, whereas pcDNA3.1‐ QKI‐5 group was inhibited by CCK‐8 assay. *P < .05, compared with NC group, ^#^ *P* < .05, compared with miR‐9‐5p mimics group, ^&&^ *P* < .01, compared with pcDNA3.1‐MEG3 group. E, F, The cell apoptosis rate of miR‐9‐5p mimics group was decreased, whereas pcDNA3.1‐QKI‐5 group was increased by FCM assay. *P < .05, **P < .01, compared with NC group, ^#^ *P* < .05, ^##^ *P* < .01, compared with miR‐9‐5p mimics group, ^&&^ *P* < .01, compared with pcDNA3.1‐ QKI‐5 group. G, H, Cell invasion ability of miR‐9‐5p mimics group was promoted, whereas pcDNA3.1‐QKI‐5 group was inhibited by transwell assay. *P < .05, compared with NC group, ^#^ *P* < .05, ^##^ *P* < .01, compared with miR‐9‐5p mimics group, ^&^ *P* < .05, compared with pcDNA3.1‐QKI‐5 group. Scale bar = 20 μm. I, QKI‐5 expression level of miR‐9‐5p mimics group was decreased, whereas pcDNA3.1‐QKI‐5 group was increased detected by Western blot. **P < .01, compared with NC group, ^##^ *P* < .01, compared with miR‐9‐5p mimics group. ^&&^ *P* < .01, compared with pcDNA3.1‐QKI‐5 group

MiR‐9‐5p was found to have targeting relations with *QKI‐5* by miRBase predictor. And then, the targeting relationship between miR‐9‐5p and QKI‐5 was also showed in Figure 4A. The luciferase intensity of DU 145 cells indicated that cotransfected with *QKI‐5* wild‐type and miR‐9‐5p was significantly reduced, whereas *QKI‐5* mutant type was hardly changed based on luciferase reporter assay (Figure 4B). Therefore, the interaction between miR‐9‐5p and *QKI‐5* was verified. The results of qRT‐PCR verified that *QKI‐5* expression level of pcDNA3.1‐QKI‐5 group was significantly promoted than other 3 groups, whereas miR‐9‐5p mimics group was significantly reduced. Besides, *QKI‐5* expression level of miR‐9‐5p mimics+pcDNA3.1‐QKI‐5group was the same as NC group (Figure 4C). CCK‐8 assay showed that the miR‐9‐5p mimics group exhibited the highest cell proliferative capacity, whereas cell activity of pcDNA3.1‐QKI‐5 group was significantly inhibited. Cell proliferative capacity of miR‐9‐5p mimics+pcDNA3.1‐QKI‐5 group was the same as NC group (Figure 4D). The results of FCM assay revealed that cell apoptosis rate of miR‐9‐5p mimics group was significantly suppressed, whereas pcDNA3.1‐QKI‐5 group was increased. And cell apoptosis rate of miR‐9‐5p mimics+pcDNA3.1‐QKI‐5 group was that same as NC group (Figure 4E,F). After transwell assay, cell invasion ability of miR‐9‐5p mimics group was the highest, whereas that of pcDNA3.1‐ QKI‐5 group was inhibited. And that of pcDNA3.1‐MEG3 & miR‐9‐5p mimics group was the same as NC group (Figure 4G,H). *QKI‐5* expression level of miR‐9‐5p mimics group was significantly decreased, whereas that of pcDNA3.1‐MEG3 group was increased detected by Western blotting (Figure 4I). As a result, MEG3 served as a sponge of miR‐9‐5p to regulate *QKI‐5*.

### 3.5 MEG3 inhibited prostate cancer in vivo

**Figure 5:**
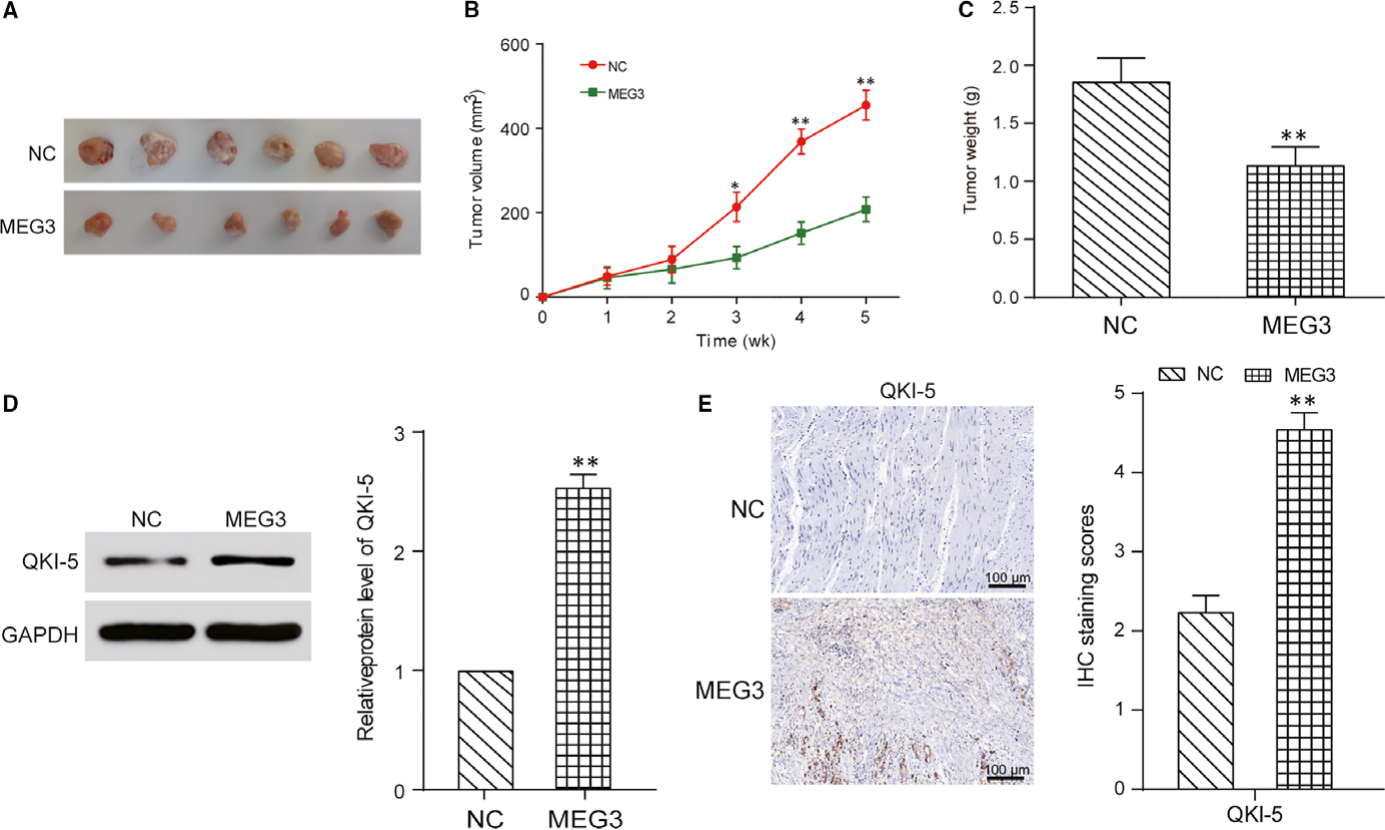
MEG3 inhibited prostate cancer in vivo. A, Photos of prostate tumour tissues after 5 weeks. B, The tumour size was smaller in the MEG3 group. *P < .05, **P < .01, compared with NC group. C, The tumour weight was lighter in the MEG3 group. **P < .01, compared with NC group. D, MEG3 overexpression increased expression level of *QKI‐5* after 5 weeks detected by Western blot. **P < .01, compared with NC group. E, MEG3 overexpression increased expression level of *QKI‐5* after 5 weeks detected by immunohistochemical analysis. **P < .01, compared with NC group. Scale bar = 100 μm

To verify the oncogenic role of MEG3 in prostate tumorigenesis, a xenograft mouse model was constructed. Overexpression MEG3 decreased tumour size after 3 weeks results compared with NC group (Figure 5A‐C). According to Western blot results, the MEG3 overexpression group exhibited higher *QKI‐5* expression than that in NC group After 5 weeks (Figure 5D). Moreover, immunohistochemical analysis revealed that MEG3 overexpression increased expression level of *QKI‐5* (Figure 5E). Collectively, these results indicated that MEG3 inhibits prostate cancer in vivo.

## 4 DISCUSSION

We screened the down‐regulated lncRNA MEG3 in prostate cancer firstly. And then, based on investigating the relationship among MEG3, miR‐9‐5p and *QKI‐5* proved that MEG3 inhibit the expression of miR‐9‐5p, whereas miR‐9‐5p down‐regulated *QKI‐5*.

LncRNAs involved in many cancer‐related research mechanisms.^2^ For example it is proved that SNHG1 is up‐regulated in lung cancer cells lines and in vitro experiments and down‐regulation of SNHG1 suppresses lung cancer cell proliferation.^10^ A plenty of lncRNAs attracted the attention of many scientists, proved that several lncRNAs involved in the regulatory mechanism of PCa, including lncRNA PCGEM1, lncRNA SOCS2‐AS1, lncRNA CTBP1‐AS, lncRNA DRAIC and lncRNA PCAT29.^2^ Xu et al^5^ found that PCAT‐1 promoted proliferation, migration, invasion and repressed apoptosis of PCa cells. What's more, it's also been reported that up‐regulation of MEG3 in prostate cancer cell lines induces cell apoptosis and G0/G1 phase arrest.^16^ The results of our study focused on detecting the effect of lncRNA MEG3 in PCa, we revealed that MEG3 was a down‐regulated lncRNA in PCa and it played a role of anti‐cancer factor.

Mounting evidence suggested that lncRNAs may exert functions by targeting miRNAs.^17^ Bian et al confirmed that UCA1 worked as a ceRNA in colorectal cancer, regulated the expression of miR‐204‐5p and induced the resistance to 5‐FU in CRC.^18^ He et al^9^ found another lncRNA that regulated cardiac hypertrophy by targeting miRNA‐489, named as CHRF. Moreover, a recent study revealed that lncRNA SNHG1 could promote cell progression by antagonizing miR‐199a‐3p in prostate cancer.^10^ Also, it has been found that MEG3 exerts its function through negatively regulating miR‐9 expression by directly interaction in vascular endothelial cells.^19^ In present study, we also found that MEG3 functions as a ceRNA for miR‐9‐5p in prostate cancer, which indicated that a mutual antagonism between MEG3 and miR‐9‐5p on impacting prostate cancer progression. This is useful for understanding the molecular mechanisms associated with prostate cancer.

Relevant studies have revealed the interactions among lncRNA, miRNA and target genes in investigation of tumour mechanism. For example Nie et al showed that lncRNA UCA1 functioned as an oncogene in NSCLC, acting mechanistically by up‐regulating *ERBB4* in part through ‘sponging’ miR‐193a‐3p.^20^ PCA3 is relieved sponging of miR‐1261, further led to suppression of the target gene *PRKD3*, and thus inhibited cell proliferation of PCa.^9^ There were studies revealed that an up‐regulated lncRNA GAS5 inhibited the pathogenesis and progression of PCa through directly targeting miR‐103, and negatively mediated the AKT/mTOR signalling pathway.^21^ In addition, published research reported that MEG3 inhibited the progression of gastric cancer through regulating miR‐181 family and its downstream targeting molecule *Bcl‐2*.^22^ We revealed a result of MEG3 functioned as a ceRNA for miR‐9‐5p to regulate *QKI‐5*, which was consistent with the previous reports. A report proved that MEG3 could regulate expression of *E‐cadherin* and *FOXO1* by competitively binding miR‐9 in oesophageal squamous cell carcinoma.^11^

Our findings revealed that overexpression of MEG3 and QKI‐5 inhibited the progression of PCa, whereas overexpression of miR‐9‐5p presented an inverse effects. However, there are also shortcomings in our research, for example the number of samples we used for this study was not large enough. In this study, we focused on *QKI‐5*, which is just a member of QKI alternatively spliced mRNAs. Therefore, the further research on functions and mechanisms underlying of other QKI genes was needed to be carried out. In addition, there was not only one targeted miRNA have potential targeting relationship with MEG3 or *QKI‐5*. However, we just focused on one targeting miRNA in this study.

## 5 CONCLUSION

In conclusion, our study showed that MEG3 was a down‐regulated lncRNA in human prostate cancer, and it could regulate miR‐9‐5p/*QKI‐5* expression. We also demonstrated that overexpression of MEG3 could effectively inhibit the development of PCa through targeting miR‐9‐5p/*QKI‐5* axis in prostate cancer. Our findings provided novel evidences that lncRNAs acted as “microRNA sponges” in prostate cancer.

## CONFLICT OF INTEREST

The authors confirm that there are no conflicts of interest.

## AUTHOR CONTRIBUTIONS

Meng Wu and Yawei Huang participated in research conception and design. Tongchang Chen and Weichao Wang involved in data analysis and interpretation. Shiguang Yang and Zhenfeng Ye performed statistical analysis. Meng Wu and Xiaoqing Xi carried out drafting of the manuscript. All authors contributed to the critical revision and approval of final manuscript.

## ETHICAL APPROVAL

All procedures involving humans was approved by the Ethics Committee of the Second Affiliated Hospital of Nanchang University and all participants signed the informed consent.

The animal experiment was performed in compliance with the authenticated animal protocols of Ethical Committee of Animal Welfare of the Second Affiliated Hospital of Nanchang University.

## INFORMED CONSENT

Informed consent has been obtained from each patient or case after full explanation of the purpose and nature of all procedures used.
